# Evaluation of the Allplex HPV assay’s adherence to international guidelines for cervical cancer screening in clinician-collected samples

**DOI:** 10.1128/spectrum.00332-24

**Published:** 2024-06-25

**Authors:** Pui Yan Jenny Chung, Sharonjit K. Dhillon, Selina Cortoos, Hannelore Hamerlinck, Rita Pereira, Elizaveta Padalko, Davy Vanden Broeck, Marc Arbyn

**Affiliations:** 1 Unit of Cancer Epidemiology, Belgian Cancer Centre, Sciensano, Brussels, Belgium; 2 Department of Molecular Diagnostics, Sonic Healthcare Benelux – AML, Antwerp, Belgium; 3 Department of Medical Microbiology, Ghent University Hospital, Ghent, Belgium; 4 Department of Diagnostics Sciences, Faculty of Medicine and Health Sciences, Ghent University, Ghent, Belgium; 5 International Centre for Reproductive Health, Ghent University, Ghent, Belgium; 6 National Reference Centre for HPV, Brussels, Belgium; 7 Department of Human Structure and Repair, Faculty of Medicine and Health Sciences, Ghent University, Ghent, Belgium; National Taiwan University, Taipei, Taiwan

**Keywords:** HPV genotyping, cervical cancer, human papillomavirus, test validation, reproducibility, clinical accuracy

## Abstract

Clinically validated human papillomavirus (HPV) assays are crucial in cervical cancer screening. In this study, we evaluated the Allplex HPV HR Detection assay (Seegene, SouthKorea) for its clinical accuracy and reproducibility according to the international criteria, using the RealTime High Risk HPV m2000 assay (Abbott, USA) as standard comparator. The Allplex HPV HR assay exhibits significant non-inferior sensitivity to detect cervical intraepithelial neoplasia grade (CIN) 2 or worse (CIN2+) with a ratio of 1.00 (95% CI: 0.97–1.03, *P* = 0.006), insignificant non-inferior sensitivity to detect CIN3+ with a ratio of 1.00 (95% CI: 0.88–1.13, *P* = 0.098), and non-inferior specificity to exclude CIN2+ with a ratio of 0.99 (95% CI: 0.99–1.00, *P* < 0.001) compared to the standard comparator. In addition, the assay shows an excellent reproducibility within the same laboratory [96.5% (95% CI: 94.6–97.9) with a kappa value of 0.91 (95% CI: 0.87–0.95)] and between laboratories [96.7% (95% CI: 94.8–98.0) with a kappa value of 0.91 (95% CI: 0.87–0.95)] for overall high-risk HPV positivity as well as for each individual HPV type. Pooling our study data with those of another independent study supports the consistency of our findings. We conclude that both the clinical accuracy to detect cervical precancer and the reproducibility of Allplex HPV HR Detection assay fulfill the international validation criteria of use in cervical cancer screening.

**IMPORTANCE:**

The clinical validation of human papillomavirus (HPV) assays in accordance with well-established international guidelines is crucial to ensure that only validated assays are used in the context of screening (Meijer et al., Int J Cancer, 2009). The guidelines, developed by an international consortium, require that a novel HPV assay has non-inferior accuracy against a standard comparator test for the detection of cervical intraepithelial neoplasia grade (CIN) 2 or worse (CIN2+). Additionally, a new HPV assay should meet specific criteria for both intra- and inter-laboratory reproducibility to ensure the assay consistently exhibits technical precision and robust performance. Pooling our study data with those of another independent study supports the consistency of our findings. In conclusion, both the clinical accuracy to detect cervical precancer and the reproducibility of Allplex HPV HR Detection assay fulfill the international validation criteria of use in cervical cancer screening.

## INTRODUCTION

Human papillomavirus (HPV) infection is a well-established condition for the development of cervical cancer (CC) (1). Nearly all CC cases are associated with high-risk (hr) HPV infection, particularly HPV16 and HPV18, which together contribute to over 70% of all CC cases (2–4). Although HPV infection is the most common viral infection of the reproductive tract, 90% of infections resolve spontaneously. However, persistent HPV infection carries a risk of progression to CC with the disease typically evolving over an extended timeframe, taking 15–20 years for CC to develop. The slow disease progression consequently makes screening an effective public health intervention that enables timely detection and early intervention. Despite an increasing global effort to promote HPV vaccination among young cohorts, CC screening continues to be a cornerstone of public health initiative in alleviating the CC burden in women aged 30 and above.

Evidence from large-scale randomized clinical trials clearly shows that hrHPV-based screening significantly reduces the incidence and mortality of CC compared to traditional cytology-based screening (5–7). The World Health Organization currently recommends HPV nucleic acid amplification tests as a first-choice screening method for CC (8). A 2020 review revealed over 250 HPV assays available on the market with over 90% of the assays lacking adequate evaluation and clinical validation (9).

Clinical validation of HPV assays in accordance with well-established international guidelines is crucial to ensure that only validated assays are used in the screening context (10). The guidelines, developed by an international consortium, require that a novel HPV assay has non-inferior accuracy against a standard comparator test (SCT) for the detection of cervical intraepithelial neoplasia grade (CIN) 2 or worse (CIN2+). Additionally, a new HPV assay should meet specific criteria for both intra- and inter-laboratory reproducibility to ensure the assay consistently exhibits technical precision and robust performance.

The Allplex HPV HR Detection assay (Seegene, Seoul, South Korea) (referred as *“AllplexHPVHR”*) is a novel multiplex real-time fluorescence polymerase chain reaction (PCR) assay that detects 14 hrHPV types. This study set out to evaluate the clinical accuracy and reproducibility of AllplexHPVHR, with cervical cell samples originating from primary CC screening in Belgium. Recently, Oštrbenk et al. (11) reported a clinical accuracy and reproducibility study with AllplexHPVHR in a Slovenian CC screening population, using a different comparator [i.e.,Hybrid Capture 2 (HC2, Qiagen, USA)]. We have extracted data from this paper and performed a meta-analysis on the relative accuracy of AllplexHPVHR.

## MATERIALS AND METHODS

### Study design and study population

The Meijer guidelines are a study protocol to validate HPV assays for the use in CC screening (10). According to this guideline, an HPV assay should fulfill the accuracy and reproducibility (intra- and inter-laboratory) criteria using samples that are representative for a screening population. In our study, we used remnant material of cervical cells from women participating in primary CC screening in Belgium. The clinician-collected samples were stored according to the manufacturer’s instructions in liquid-based cytology medium (PreservCyt, Hologic, Marlborough, MA, USA) at the Sonic Healthcare Benelux (AML) biobank.

For the clinical accuracy assessment, 860 samples were included in a panel (referred as *“AML_Meijer_1”*) of 60 CIN2+ cases (of which 16 cases are confirmed CIN3+) and 800 cytological and viral negative controls; all selected according to the Meijer criteria (10) and from a population-based screening cohort. The 60 CIN2+ samples were randomly selected out of a pool of cervical specimens from women with a histologically confirmed CIN2+ result. HPV positivity was determined using the Riatol qPCR assay (12) as a pre-selection. Viral loads were not considered for the selection of the 60 CIN2+ histology-positive samples, only Ct-values were documented. Controls were randomly selected out of a pool of cervical specimens from women of at least 30 years of age. These women had a documented negative HPV test result (12) and a prior negative cytology screening result no more than 3 years before.

To assess the intra- and inter-laboratory reproducibility, 550 screening samples were selected from the AML biobank for the reproducibility panel (referred as *“AML_Repro_1”*). The 550 samples were tested using the Abbott RealTime High Risk HPV m2000 assay (Abbott, Chicago, IL, USA) (referred as *“RealTime”*). The AML_Repro_1 panel includes 176 RealTime-positive samples and 374 RealTime-negative samples, according to validation guidelines of including at least 30% HPV-positive samples (10).

### Index test

The index test was the Seegene AllplexHPVHR assay, an innovative qualitative real-time PCR-based assay that incorporates patented technologies such as Dual-Priming Oligonucleotides (DPO), Tagging Oligonucleotide Cleavage and Extension (TOCE), Multiple Detection Temperature (MuDT), and “3 Ct” to allow simultaneous detection of multiple targets per fluorophore. AllplexHPVHR targets the *E6/E7* region for DNA detection of HPV 16 and HPV 18, and the *L1* gene for DNA detection of 12 other hrHPV types (i.e., HPV 31, 33, 35, 39, 45, 51, 52, 56, 58, 59, 66, and 68). The *beta-globin* gene is added as an internal control. Targets are detected separately in five channels with amplification of fluorescent signals (HEX, FAM, Cal Red 610, Quasar 670, and Quasar 705), providing Ct values as output. Analysis is done with Seegene Viewer (V3.30.000, trial version) software. Positivity and negativity of a sample were determined according to the manufacturer’s instructions: the Ct threshold of the internal control is ≤43, the Ct threshold of HPV 16 and 18 is ≤40, the Ct threshold of HPV 31, 33, 45, 52, and 58 is ≤37, and the Ct threshold of HPV 35, 39, 51, 56, 59, 66, and 68 is ≤35.

### Standard comparator test

The SCT was the Abbott RealTime High Risk HPV m2000 assay, which is a second-generation standard comparator (communicated at EUROGIN—International Multidisciplinary HPV conferences in 2023 and 2024 and at the 35th International Papillomavirus Conference 2023). Since the standard comparator hrHPV tests (HC2 and GP5+/6+) are infrequently used in current clinical practice and lack genotyping capabilities, newer hrHPV DNA comparator tests have been proposed, and RealTime has been added to the list of alternative hrHPV DNA comparator tests (13). RealTime is an automated multiplex PCR, targeting the *L1* gene. It detects 14 hrHPV genotypes and has been consistently validated in several previous studies (14–16). RealTime allows separate genotyping of HPV 16 and HPV 18 and an aggregate of 12 other hrHPV genotypes (HPV 31, 33, 35, 39, 45, 51, 52, 56, 58, 59, 66, and 68) (15).

### Clinical accuracy testing

Accuracy criteria testing was carried out at AML, and the panel was tested once with the SCT and once with the index test according to the manufacturer’s recommendations. Briefly, all primary samples from the AML_Meijer_1 panel were extracted in batches of 92 samples using the Seegene STARMag96 Universal Extraction System on the fully automated Seegene STARlet instrument according to the manufacturer’s instructions for AllplexHPVHR. For PCR amplification, a Bio-Rad CFX96 instrument was used in combination with the Seegene Viewer (V3.30.000, trial version) for subsequent HPV genotyping interpretation and clinical cut-off implementation. According to the manufacturer, other compatible DNA extraction and/or PCR instruments—not used in this study—are SEEPREP32 (an extraction-only system), Seegene STARlet-AIO (an all-in-one automated extraction and PCR system), Seegene STARlet 96MPH, and Seegene NIMBUS (the latter two are automated extraction and PCR setup systems). In parallel, the SCT was performed on the same primary samples, which were first extracted with the Abbott mSample Preparation System on the fully automated Abbott m2000sp instrument. PCR amplification was performed on the Abbott m2000rt instrument, and results were compared to the AllplexHPVHR results.

### Reproducibility testing

To assess the AllplexHPVHR reproducibility, three aliquots of remnant primary material were obtained from each sample where aliquots 1 and 2 were tested with AllplexHPVHR within the same laboratory (AML) for the intra-laboratory assessment (run 1 versus run 2). Run 1 was carried out on 3 and 4 April 2023 and run 2 on 5 and 6 April 2023. The third aliquot was sent to another laboratory (Laboratory of Clinical Biology, UZ Ghent) for inter-laboratory reproducibility (assessed between 3 and 7 April 2023); whereafter, the results were compared to the run 1 results obtained from AML. Briefly, all primary samples from the AML_Repro_1 panel followed the same sample handling procedure for AllplexHPVHR at AML as with the AML_Meijer_1 panel. This procedure was carried out twice at AML for the intra-laboratory assessment. At UZ Ghent, samples were maintained at 4°C until processing, involving a brief spin and the transfer of 300 µL into an extraction tube (comprising 100 µL dead volume). Nucleotide extraction was executed using the STARMag96 × 4 Universal Cartridge Kit, with an elution volume of 100 µL. Subsequently, 5 µL of DNA extract was employed in the AllplexHPVHR protocol, utilizing default parameters.

### Statistical analyses

Clinical performance of AllplexHPVHR was evaluated in terms of absolute sensitivity for CIN2+ and specificity for controls, with 95% CI. When the +/− and −/+ results were zero, CIs were computed by adding 0.5 to each cell of the contingency table. To determine the non-inferiority of the index test compared to SCT, we followed Tang’s proposed method, which uses benchmarks of 0.90 for relative sensitivity and 0.98 for relative specificity.

Reproducibility was measured as the overall percentage agreement, which indicates the proportion of concordant results (i.e., both tests being positive or both negative) out of all results. The reproducibility criterion was considered met if the lower bound of the 95% CI for concordance of all hrHPV types and of each hrHPV type separately exceeded 87%, and if the kappa (κ) statistic was >0.5.

Analytical concordance between AllplexHPVHR and RealTime was assessed using kappa statistics, for HPV 16 and HPV 18 as well as for the grouped 12 hrHPV types. Kappa values were categorized as excellent (1.00 ≥ κ > 0.80), good (0.80 ≥ κ > 0.60), moderate (0.60 ≥ κ > 0.40), fair (0.40 ≥ κ > 0.20), or poor (0.20 ≥ κ > 0.00).

Meta-analysis of relative sensitivity and specificity of AllplexHPVHR to comparator was performed using a random effects model for pooling ratios of proportions, and a forest plot was constructed (17). Non-inferiority was confirmed when the left 90% CI bound around the relative accuracy exceeds 0.90 (for sensitivity) or 0.98 (for specificity).

Furthermore, 95% exact CIs were computed for all proportions, considering the paired design in comparisons when calculating confidence intervals for relative sensitivity and specificity. Statistical analyses were conducted using STATA version 16 (College Station, TX, USA).

## RESULTS

### Clinical performance

The internal controls of both AllplexHPVHR and RealTime were amplified yielding 860 valid samples for the clinical accuracy analyses. For the clinical sensitivity analysis, AllplexHPVHR yielded a positive result for 56/60 CIN2+ cases, resulting in an absolute clinical sensitivity of 93.3% (95% CI: 87.0–99.6). RealTime also showed 56/60 positive results for CIN2+ showing identical results of absolute clinical sensitivity. The relative sensitivity of AllplexHPVHR was 1.00 with a *P*-value for non-inferiority (*P*_ni_) of 0.006, indicating non-inferior clinical sensitivity of AllplexHPVHR compared to RealTime. For the identification of CIN3 cases, both assays detected 15/16 CIN3 cases and missed 1 CIN3 case. For controls, AllplexHPVHR yielded negative results for 795/800 samples [absolute specificity: 99.4% (95% CI: 98.8–99.9)], whereas RealTime was negative for 798/800 controls [absolute specificity: 99.8% (95% CI: 99.4–100.0)]. AllplexHPVHR was non-inferior for specificity compared to RealTime (*P*_ni_ < 0.001). The clinical performance estimates are shown in Table 1. Out of the 56 concordantly positive CIN2+ cases detected by both AllplexHPVHR and RealTime, 14 were single HPV 16 infections, 3 were single HPV 18 infections, 26 were other infections not containing HPV 16 or HPV 18, and 13 were multiple infections (Table 2). In Table 3, we describe the prevalence of type-specific HPV infections, determined by AllplexHPVHR among CIN2+ cases in two rankings: (i) non-hierarchical and (ii) hierarchical. The hierarchical ranking is according to the risk of cervical cancer associated with each HPV type (3). HPV 16, the most potent carcinogenic type, was the most frequently detected among the CIN2+ cases, accounting for 44.6% (25/56). HPV 18, the second most potent carcinogenic type, was found in seven cases (12.5%), with two of these cases co-infected with HPV 16. HPV 16 or 18 was collectively present in 30 cases (53.6%). Additionally, in 50 cases (89.3%), one or more HPV types included in the nonavalent vaccine were detected (Table 3).

**TABLE 1:** Absolute values allowing the computation of the accuracy of AllplexHPVHR compared to RealTime in the clinical accuracy panel

AllplexHPVHR		RealTime		
		Positive	Negative	Total
Contingency matrix for the assessment of the sensitivity for CIN2+^*a*^				
	Positive	56	0	56
	Negative	0	4	4
	Total	56	4	60
Contingency matrix for the assessment of the specificity for controls^*b*^				
	Positive	1	4	5
	Negative	1	794	795
	Total	2	798	800

^*a*^ Relative sensitivity: 1.000 (95% CI: 0.966–1.035); *P*_non-inferiority_ = 0.006.

^*b*^ Relative specificity: 0.996 (95% CI: 0.991–1.002); *P*_non-inferiority_< 0.001.

**TABLE 2:** Single and multiple infections among the 56 concordantly hrHPV-positive AllplexHPVHR + /RealTime + CIN2+ cases^*a*^

AllplexHPVHR	RealTime						
	HPV 16+ alone	HPV 16+ and 18+	HPV 16+ and other+	HPV 16+, HPV 18+ and other+	HPV 18+ alone	HPV 18+ and other+	Other+ alone
HPV 16+ alone	14	0	0	0	0	0	0
HPV 16+ and 18+	0	0	0	0	0	0	0
HPV 16+ and other+	3	0	5	0	0	0	1
HPV 16+, HPV 18+ and other+	0	1	0	1	0	0	0
HPV 18+ alone	0	0	0	0	3	0	0
HPV18+ and other+	0	0	0	0	0	2	0
Other+ alone	0	0	0	0	0	0	26

^*a*^ Other: HPV 31/33/35/39/45/51/52/56/58/59/66/68.

**TABLE 3:** Non-hierarchical and hierarchical ranked genotypes by AllplexHPVHR in the CIN2+ cases

AllplexHPVHR^*a*^	CIN2+	
	Non-hierarchical^*b*^ [*n* (%)]	Hierarchical^*c*^ [*n* (%)]
HPV 16	25 (44.6%)	25 (44.6%)
HPV 18	7 (12.5%)	30 (53.6%)
HPV 45	3 (5.4%)	32 (57.1%)
HPV 33	9 (16.1%)	38 (67.9%)
HPV 58	8 (14.3%)	43 (76.8%)
HPV 31	9 (16.1%)	49 (87.5%)
HPV 52	5 (8.9%)	50 (89.3%)
HPV 35	1 (1.8%)	51 (91.1%)
HPV 59	2 (3.6%)	51 (91.1%)
HPV 39	2 (3.6%)	51 (91.1%)
HPV 68	4 (7.1%)	51 (91.1%)
HPV 51	10 (17.9%)	55 (98.2%)
HPV 56	1 (1.8%)	56 (100.0%)
HPV 66	4 (7.1%)	56 (100.0%)

^a^ HPV types are ranked according to their level of carcinogenicity (3).

^b^ Non-hierarchical: Samples with all HPV types are counted, irrespective of single or multiple infections.

^c^ Hierarchical: Samples with a single and a multiple infection of the most carcinogenic HPV type (HPV 16) are counted first, followed by adding samples that contain single infection with the second-most carcinogenic HPV type (HPV 18) on top of HPV 16 (single and multiple infection) and so forth (in an ascending way).

### Intra- and inter-laboratory reproducibility

Of the 550 samples in the reproducibility panel, five samples had a Ct value >43 for the internal control when tested with AllplexHPVHR. These five invalid samples were excluded. A total of 545 samples were included for the reproducibility assessment. In the intra-laboratory testing, there were 526/545 concordant samples, and the overall hrHPV agreement was 96.5% (95% CI: 94.6–97.9) with κ = 0.91 (95% CI: 0.87–0.95) (Table 4). The inter-laboratory assessment exhibited 527/545 concordant samples and an overall agreement of 96.7% (95% CI: 94.8–98.0) and κ = 0.91 (95% CI: 0.87–0.95) (Table 4).

**TABLE 4:** Intra- and inter-laboratory reproducibility of AllplexHPVHR

		AML run 1		
		Negative	Positive	Total
Intra-laboratory analysis^*a*^				
AML run 2				
	Negative	387	5	392
	Positive	14	139	153
	Total	401	144	545
Inter-laboratory analysis^*b*^				
UZ Ghent run				
	Negative	392	9	401
	Positive	9	135	144
	Total	401	144	545

^*a*^ Overall intra-laboratory hrHPV test agreement: 96.5 % (95% CI: 94.6%–97.9%); Kappa: 0.912 (95% CI: 0.873–0.951).

^*b*^ Overall inter-laboratory hrHPV test agreement: 96.7 % (95% CI: 94.8%–98.0%); Kappa: 0.915 (95% CI: 0.877–0.954).

Additionally, the reproducibility of AllplexHPVHR at the genotype-specific level—both in the intra- and inter-laboratory analysis—consistently demonstrated an overall agreement between 97% and 99% for HPV 16 and the 10 other hrHPV types and 100% for HPV 18, 35, and 58 (Tables 5 and 6). The kappa values for all the specific genotypes were higher than 0.80. The intra-laboratory general agreement of the hrHPV types separately ranges from 99.3% to 100.0% (κ = 0.83–1.00), indicating an excellent intra-laboratory reproducibility of each hrHPV type (Table 5). Similar results are found in the inter-laboratory general agreement (99.3%–100.0%) and the kappa value of each hrHPV type (0.80–1.00) (Table 6).

**TABLE 5:** Genotype-specific level agreement of the AllplexHPVHR assay for intra-laboratory analysis

HPV type	−/−^*a*^	+/+^*a*^	−/+^*a*^	+/−^*a*^	General agreement (95% CI)	Kappa (95% CI)
Intra-laboratory analysis						
HPV 16	514	25	3	3	98.9% (97.6%–99.6%)	0.89 (0.80–0.98)
HPV 18	538	7	0	0	100.0% (99.3%–100.0%)^*c*^	1.00 (1.00–1.00)
Other hrHPV^*b*^	414	118	11	2	97.6% (96.0%–98.7%)	0.93 (0.90–0.97)
HPV 31	525	19	1	0	99.8% (99.0%–100.0%)	0.97 (0.92–1.00)
HPV 33	530	11	3	1	99.3% (98.1%–99.8%)	0.84 (0.69–0.99)
HPV 35	539	6	0	0	100.0% (99.3%–100.0%)^*c*^	1.00 (1.00–1.00)
HPV 39	531	14	0	0	100.0% (99.3%–100.0%)^*c*^	1.00 (1.00–1.00)
HPV 45	530	12	3	0	99.4% (98.4%–99.9%)	0.89 (0.76–1.00)
HPV 51	531	13	1	0	99.8% (99.0%–100.0%)	0.96 (0.89–1.00)
HPV 52	520	23	2	0	99.6% (98.7%–100.0%)	0.96 (0.90–1.00)
HPV 56	531	11	1	2	99.4% (98.4%–99.9%)	0.88 (0.74–1.00)
HPV 58	532	13	0	0	100.0% (99.3%–100.0%)^*c*^	1.00 (1.00–1.00)
HPV 59	535	8	1	1	99.6% (98.7%–100.0%)	0.89 (0.73–1.00)
HPV 66	531	13	0	1	99.8% (99.0%–100.0%)	0.96 (0.89–1.00)
HPV 68	538	5	2	0	99.6% (98.7%–100.0%)	0.83 (0.60–1.00)

^*a*^ −/−: both runs are concordantly negative; +/+: both runs are concordantly positive; −/+: run 1 negative AML, run 2 positive AML; +/−: run 1 positive AML, run 2 negative AML.

^*b*^ Other hrHPV includes the aggregate of HPV types 31, 33, 35, 39, 45, 51, 52, 56, 58, 59, 66, and 68.

^*c*^ One-sided statistics, 97.5% confidence interval.

**TABLE 6:** Genotype-specific level agreement of the AllplexHPVHR assay for inter-laboratory analysis

HPV type	−/−^*a*^	+/+^*a*^	−/+^*a*^	+/−^*a*^	General agreement (95% CI)	Kappa (95% CI)
Inter-laboratory analysis						
HPV 16	516	25	1	3	99.3% (98.1%–99.8%)	0.92 (0.85–1.00)
HPV 18	538	7	0	0	100.0% (99.3%–100.0%)^*c*^	1.00 (1.00–1.00)
Other hrHPV^*b*^	417	114	8	6	97.4% (95.7%–98.6%)	0.93 (0.89–0.96)
HPV 31	525	17	1	2	99.4% (98.4%–99.9%)	0.92 (0.82–1.00)
HPV 33	532	12	1	0	99.8% (99.0%–100.0%)	0.96 (0.88–1.00)
HPV 35	539	6	0	0	100.0% (99.3%–100.0%)^*c*^	1.00 (1.00–1.00)
HPV 39	531	13	0	1	99.8% (99.0%–100.0%)	0.96 (0.89–1.00)
HPV 45	532	12	1	0	99.8% (99.0%–100.0%)	0.96 (0.88–1.00)
HPV 51	531	13	1	0	99.8% (99.0%–100.0%)	0.96 (0.89–1.00)
HPV 52	520	21	2	2	99.3% (98.1%–99.8%)	0.91 (0.82–1.00)
HPV 56	531	11	1	2	99.4% (98.4%–99.9%)	0.88 (0.74–1.00)
HPV 58	532	13	0	0	100.0% (99.3%–100.0%)^*c*^	1.00 (1.00–1.00)
HPV 59	535	8	1	1	99.6% (98.7%–100.0%)	0.89 (0.73–1.00)
HPV 66	531	14	0	0	100.0% (99.3% - 100.0%)	1.00 (1.00–1.00)
HPV 68	539	4	1	1	99.6% (98.7% - 100.0%)	0.80 (0.52–1.00)

^*a* *−*^ /−: both runs are concordantly negative; +/+: both runs are concordantly positive; −/+: run 1 negative AML, run 2 positive UZ Ghent; +/−: run 1 positive AML, run 2 negative UZ Ghent.

^*b*^ Other hrHPV includes the aggregate of HPV types 31, 33, 35, 39, 45, 51, 52, 56, 58, 59, 66, and 68.

^*c*^ One-sided statistics, 97.5% confidence interval.

### Meta-analysis of the relative accuracy

**Fig 1:**
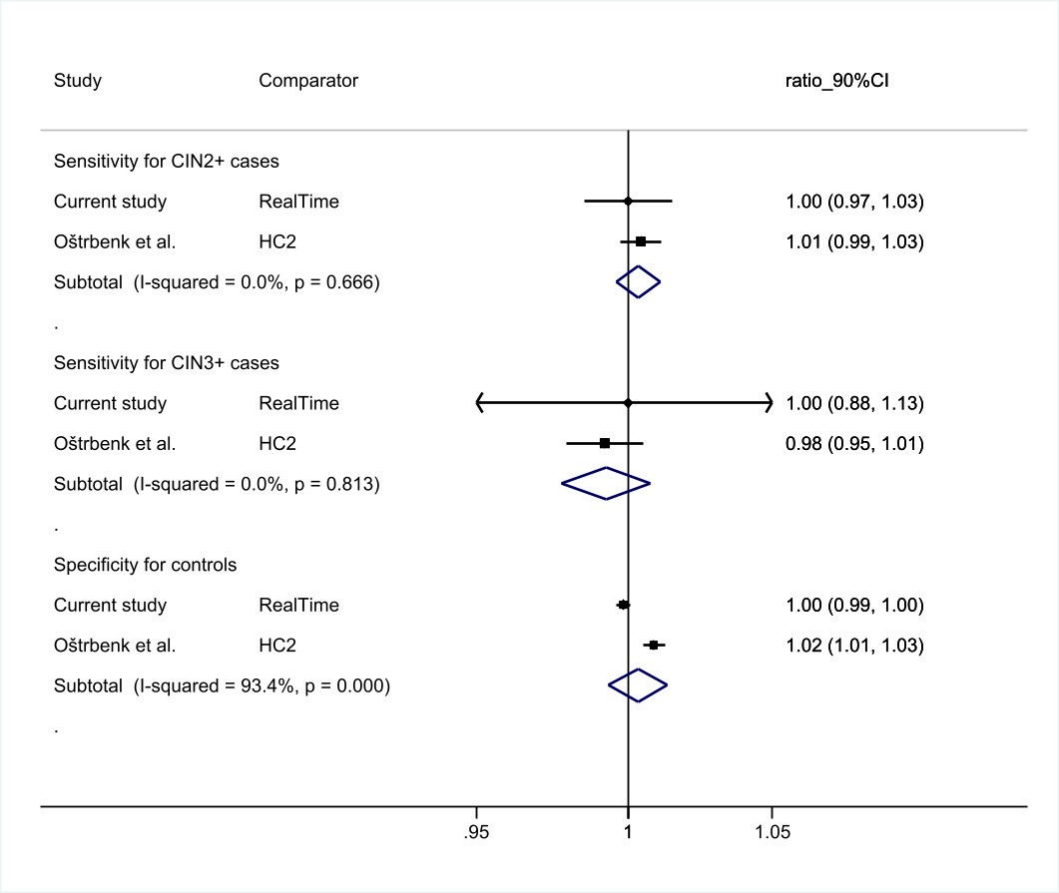
Meta-analysis of the relative sensitivity for CIN2+ (top) and CIN3+ (middle) and specificity for controls (bottom) of AllplexHPVHR versus the comparator test

We found one validation study on AllplexHPVHR conducted in Slovenia that used the standard comparator test HC2 (11). AllplexHPVHR—of which the used Ct cutoff was the same as in this study—showed a non-inferior sensitivity for CIN2+ [ratio: 1.01 (95% CI: 0.98–1.04), *P*_ni_ = 0.0004] and for CIN3+ [ratio: 0.98 (95% CI: 0.95–1.02), *P*_ni_ = 0.02], and a non-inferior specificity for controls [ratio: 1.02 (95% CI: 1.01–1.03), *P*_ni_ < 0.00001) compared to HC2. The intra- and inter-laboratory reproducibility were 98.1% (95% CI: 96.5%–99.0%) and 97.9% (95% CI: 96.3%–98.8%), respectively. Pooling our and the Slovenian study resulted in an overall relative sensitivity for CIN2+ of 1.01 (90% CI: 0.99–1.03, *P* = 0.666) and for CIN3+ of 0.98 (90% CI: 0.95–1.02, *P* = 0.813) and an overall relative specificity of 1.01 (90% CI: 0.98–1.03, *P* = 0.000) of AllplexHPVHR (Fig. 1).

## DISCUSSION

In this comprehensive validation study, AllplexHPVHR demonstrated non-inferior clinical sensitivity and specificity for the detection of cervical precancer CIN2+ compared to the SCT, and the non-inferiority for the identification of CIN3+ cases compared to the SCT was not assessed due to low number of CIN3+. Additionally, AllplexHPVHR exhibited excellent intra- and inter-laboratory reproducibility for overall results of the assay and for each hrHPV type separately. This study establishes the AllplexHPVHR assay as the fifth full genotyping HPV assay that is fully validated.

AllplexHPVHR makes use of several patented technologies from Seegene, including (i) DPO, (ii) TOCE that includes a pitcher and a catcher in the reaction, (iii) MuDT, and (iv) “3 Ct” technology. The TOCE approach makes AllplexHPVHR unique in distinguishing HPV types separately in one reaction tube, unlike a conventional real-time PCR having a set of primers and probe. The MuDT technology allows simultaneous detection of various targets in the same fluorescent channel and at multiple pre-programmed temperatures, unlike a melting temperature analysis where the temperature decreases by a gradient while reading the fluorescent signal. AllplexHPVHR is a low-cost and relatively high-throughput assay. Although the procedure of the automated STARlet system is quite complex, the integrated step-by-step real-time instructions provided by the system are user-friendly. The automated workflow from DNA extraction until obtaining PCR results takes about 4 hours. No random access is allowed, and samples can only be analyzed in batch. In an 8-hour shift, 3 runs of 92 samples can be performed with AllplexHPVHR. Similar performance is witnessed with RealTime. However, AllplexHPVHR detects 14 hrHPV types separately and simultaneously; whereas RealTime detects HPV 16, HPV 18, and the other 12 hrHPV types as aggregate. AllplexHPVHR and the STARlet system are more suitable for high-throughput laboratories than low-throughput laboratories. Maintenance and troubleshooting of the automated STARlet system require expertise and resources, which can be less suitable for low-resource setting.

Oštrbenk et al. concluded from a Slovenian study, similar to ours, that the international criteria for HPV assay validation of AllplexHPVHR were fulfilled (11). Oštrbenk et al. included 110 cases (52 CIN2, 55 CIN3, and 3 squamous cell cervical cancers) and 863 controls defined by screening with cytology and HPV assay, and a previously negative cytology screening results 3 years before. Our study independently confirmed the Slovenian findings, and the meta-analysis highlighted consistency of both validation studies. We note that there is a slight difference in the relative specificity between this study and the Slovenian study. One of the possible explanations is the selection criteria of the controls: in this study, the controls were selected based on a negative HPV test result with a prior negative cytology screening result of no more than 3 years before, while in the previous studies, the controls selection was based on cytology test results at the time of screening and the prior screening (16, 18, 19). Another possible explanation is the usage of different standard comparators: in the Slovenian study, the HC2 assay which is a signal amplification assay known to cross-react with HPV types not belonging to the IARC group I carcinogenic types, while the PCR-based Abbott RealTime assay was used in this study.

As far as we know, the Abbott RealTime HPV test is used for the first time as a stand-alone second-generation standard comparator in a clinical assessment. There are four criteria for an HPV assay to be fulfilled in becoming a second-generation SCT: (i) consistent validation of the clinical accuracy [sensitivity for both CIN2+ and CIN3+ and specificity for controls in at least three other studies against the first-generation SCTs (which includes HC2 and GP5+/6+ PCR EIA assays)]; (ii) pooled non-inferior sensitivity for CIN2+ and CIN3+ using 0.95 as benchmark compared to the first-generation SCTs; (iii) documented intra- and inter-laboratory reproducibility in at least one validation study; and (iv) target all the 12 IARC group I carcinogenic HPV types (i.e., HPV 16, 18, 31, 33, 35, 39, 45, 51, 52, 56, 58, and 59) with allowance for HPV 66 and HPV 68 (since also targeted by the first-generation SCTs). The RealTime assay was validated against HC2 in four studies (14, 15, 20, 21) and against GP5+/6+ PCR EIA in one study (16).

Limitations of our study include the study design that may be prone to selection bias in cases and controls. Despite this, the Meijer protocol is a well-established protocol and is accepted by the international scientific community as a methodology sound design to validate HPV assays. Designs such as the VALGENT protocol have been developed, where validation of HPV assays is carried out by using samples integrated into population-based, well-established screening programs, bolstered by comprehensive registries. Additionally, there is a limitation in the specificity assessment since the HPV-positive rate in the controls is lower (i.e., 0.62%) than in previous similar studies (16, 18, 19). This is explained by the selection criteria of the controls. Another limitation is that the AllplexHPVHR is a full genotyping assay, while RealTime is a partial genotyping assay that identifies HPV 16, HPV 18, and the other aggregated hrHPV types. This does not allow the comparison of the type-specific performance. So far, four full genotyping assays—the Anyplex (Seegene, Seoul, South Korea) (22), PapilloCheck (Greiner Bio-One, Frickenhausen, Germany) (23), CLART HPV4S (GENOMICA SAU, Madrid, Spain) (24), and Riatol qPCR [Sonic Healthcare Benelux (AML), Antwerp, Belgium] (25)—have been fully validated (13). However, since these assays have not yet achieved the status of standard comparator, they cannot be used in studies to analyze the type-specific performance. The relative sensitivity of AllplexHPVHR versus RealTime to detect CIN3+ cases was unity. However, the non-inferiority was not statistically significant due to limited number of CIN3+ cases in the current study. The current validation guidelines describe the clinical endpoint of CIN2+. In the future validation guidelines, CIN3+ will be included as an additional clinical endpoint since it is more prone to progress to cancer.

It is noteworthy that the role of HPV assay genotyping is assuming an increasingly important position in CC screening recommendations. Experts concur that the integration of HPV assays with extended genotyping capabilities is essential for more streamlined approaches to risk stratification in patient management (26). Such an approach serves the dual purpose of reducing the costs associated with over-screening populations and curbing unnecessary referrals. Assays with extended genotyping capabilities offer valuable insights into the changing landscape of HPV genotypes, facilitating the assessment of vaccine impact and informing public health policy decisions.

In conclusion, hrHPV testing on clinician-collected samples using AllplexHPVHR demonstrated non-inferior clinical accuracy to that of the SCT for identifying CIN2+ cases and excellent intra- and inter-laboratory reproducibility according to the international validation criteria. AllplexHPVHR fulfills all the criteria required for the clinical validation of HPV assays and should be considered validated for primary cervical cancer screening.

## Data Availability

Data sets generated by validation studies are stored locally and securely at Sciensano. Anonymized data can be made available by request to the corresponding author on a case-by-case basis pending approval from the information security coordinator at Sciensano.

## AFTER EPUB

[This article was published on 25 June 2024 with an error in Table 4. Table 4 was corrected in the current version, posted on 8 July 2024.]
